# Occurrence and Distribution of Antibiotics in the Water, Sediment, and Biota of Freshwater and Marine Environments: A Review

**DOI:** 10.3390/antibiotics11111461

**Published:** 2022-10-23

**Authors:** Zeinab Maghsodian, Ali Mohammad Sanati, Tebogo Mashifana, Mika Sillanpää, Shengyu Feng, Tan Nhat, Bahman Ramavandi

**Affiliations:** 1 Department of Environmental Science, Persian Gulf Research Institute, Persian Gulf University, Bushehr 7516913817, Iran; 2 Department of Chemical Engineering, School of Mining, Metallurgy and Chemical Engineering, University of Johannesburg, P.O. Box 17011, Doornfontein 2028, South Africa; 3 Department of Applied Physics, Faculty of Science and Technology, Universiti Kebangsaan Malaysia, Bangi 43600, Selangor, Malaysia; 4 Zhejiang Rongsheng Environmental Protection Paper Co., Ltd., NO. 588 East Zhennan Road, Pinghu Economic Development Zone, Pinghu 314213, China; 5 Department of Civil Engineering, University Centre for Research & Development, Chandigarh University, Gharuan, Mohali 140413, Punjab, India; 6 International Research Centre of Nanotechnology for Himalayan Sustainability (IRCNHS), Shoolini University, Solan 173212, Himachal Pradesh, India; 7 Institute of Research and Development, Duy Tan University, Da Nang 550000, Vietnam; 8 School of Engineering & Technology, Duy Tan University, Da Nang 550000, Vietnam; 9 Systems Environmental Health and Energy Research Center, The Persian Gulf Biomedical Sciences Research Institute, Bushehr University of Medical Sciences, Bushehr 7518759577, Iran

**Keywords:** emerging concern, antibiotics, marine environments, sediments, biota

## Abstract

Antibiotics, as pollutants of emerging concern, can enter marine environments, rivers, and lakes and endanger ecology and human health. The purpose of this study was to review the studies conducted on the presence of antibiotics in water, sediments, and organisms in aquatic environments (i.e., seas, rivers, and lakes). Most of the reviewed studies were conducted in 2018 (15%) and 2014 (11%). Antibiotics were reported in aqueous media at a concentration of <1 ng/L–100 μg/L. The results showed that the highest number of works were conducted in the Asian continent (seas: 74%, rivers: 78%, lakes: 87%, living organisms: 100%). The highest concentration of antibiotics in water and sea sediments, with a frequency of 49%, was related to fluoroquinolones. According to the results, the highest amounts of antibiotics in water and sediment were reported as 460 ng/L and 406 ng/g, respectively. In rivers, sulfonamides had the highest abundance (30%). Fluoroquinolones (with an abundance of 34%) had the highest concentration in lakes. Moreover, the highest concentration of fluoroquinolones in living organisms was reported at 68,000 ng/g, with a frequency of 39%. According to the obtained results, it can be concluded that sulfonamides and fluoroquinolones are among the most dangerous antibiotics due to their high concentrations in the environment. This review provides timely information regarding the presence of antibiotics in different aquatic environments, which can be helpful for estimating ecological risks, contamination levels, and their management.

## 1. Introduction

Today, drugs are an integral part of life and are used to treat diseases of humans and other organisms [1]. Antibiotics are substances produced or derived from an organism that kills other microorganisms and can also inhibit their growth [2]. Since the discovery of the first antibiotic by Fleming (1929), various groups of antibiotics have been identified and developed around the world and applied to treat human, animal, and plant diseases caused by pathogenic bacteria [3,4]. The discovery of antibiotics is the most remarkable scientific and medical milestone of the 20th century. The use of antibiotics in human and veterinary medicine has led to significant reductions in mortality and complications from important infectious diseases such as tuberculosis, syphilis, pneumonia, and gonorrhea [1]. Antibiotics are sometimes used as growth promoters in humans and other organisms. Their use as growth stimulants has been banned in Europe since 2006, but they are still used for this purpose in many countries, including India and China [3].

Antibiotic compounds have different mechanisms in cells, such as suppression of cell wall synthesis, inhibition of nucleic acid synthesis, modification of cell membranes, suppression of protein synthesis, and inhibition of DNA, depending on the different functions of the molecule [4].

Some antibiotic molecules are metabolized in the bodies of humans or animals, while most (70–90%) are excreted unchanged via feces and urine [5,6]. The antibiotic molecules enter wastewater as main compounds or metabolites from the effluents of hospitals, pharmaceutical companies, wastewater treatment plants (WWTPs), aquaculture, and livestock farms. The low removal capability of WWTPs has led to the transfer of large amounts of antibiotics to surface water, groundwater, and even drinking water. Marine environments are the primary locations of antibiotic accumulation [7]. The pervasive presence of antibiotics at high concentrations in surface water, groundwater, sediments, and biota worldwide has made these compounds pollutants of emerging concern [8]. Concentrations of antibiotics in both aqueous and solid-phase (e.g., biota and sediments) media have been reported as ‘ng/L and μg/L’ and ‘ng/g and μg/g’, respectively [9]. The presence of these compounds in aquatic environments—especially in developing countries where antibiotic management practices and antibiotic-related wastes have not been effectively addressed—raises further concerns [10].

The accumulation of antibiotics in different parts of aquatic environments threatens the relevant ecosystems and affects the health of humans and other organisms. Antibiotic residues in marine ecosystems cause the spread of antibiotic-resistance genes, and also lead to serious environmental problems. Connecting antibiotics to pathogens and transferring them to the human body (e.g., during the consumption of water or living organisms contaminated with antibiotics) can cause severe human risks [11]. The presence of antibiotics in aquatic environments affects the growth and reproduction of marine organisms, and also causes liver toxicity to marine organisms [10]. Antibiotic residues in the environment put pressure on the bacterial population and, eventually, cause resistant bacteria, even at low concentrations below inhibition [12]. The use of antibiotics has been increasing day by day in recent years. According to studies, the consumption of antibiotics is about 100,000 to 200,000 tons per year [13]. The level of antibiotic use increased by 65% from 2000 to 2015. Antibiotic use is also projected to increase by 200% by 2030 [14].

Recently, due to the high production and use of antibiotics and the emergence of antibiotic-resistance genes in aquatic environments, the attention of researchers around the world has been drawn to this issue [15]. To improve the current state of knowledge about the transport routes, fates, and effects of antibiotics with respect to the environment, it is essential to determine the levels of antibiotic contamination in aquatic environments [16]. The information about the environmental behaviors, fates, and adverse effects of antibiotics in water, sediments, and biota is essential for establishing legal supervision frameworks for antibiotic misuse, water quality criteria, and discharging standards for antibiotics. In recent years, many studies have been conducted on antibiotics in aquatic environments—especially in China [9,17,18,19]—but in general, information on antibiotics in such environments is not profoundly reviewed. There are a handful of review articles on the occurrence, fates, sources, and hazards of antibiotics in global waters [8,20]. Considering that antibiotics have been observed in different environments—including groundwater [21], lakes [22], surface water [23], soils [24], and sewage [25]—and due to the threat posed by antibiotics to human health and other organisms [26], reviewing the latest published works on antibiotics is informative and important. Accordingly, this study was designed to review the occurrence of antibiotics in different parts of marine environments (i.e., water, sediments, and biota) worldwide. In addition, the present review aimed to present the concentrations of antibiotics in sediments, water, and biota in water bodies worldwide and investigate the spatial distribution of antibiotics in the mentioned environments.

## 2. Methodology

**Figure 1:**
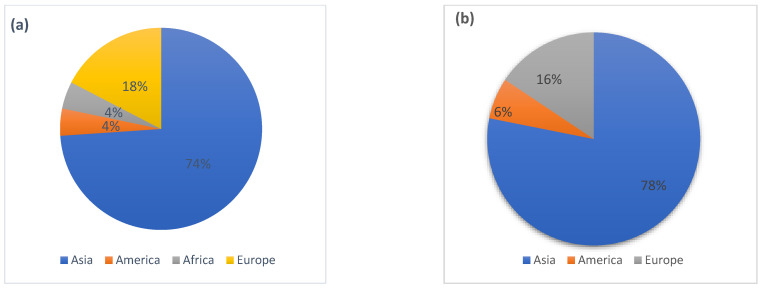

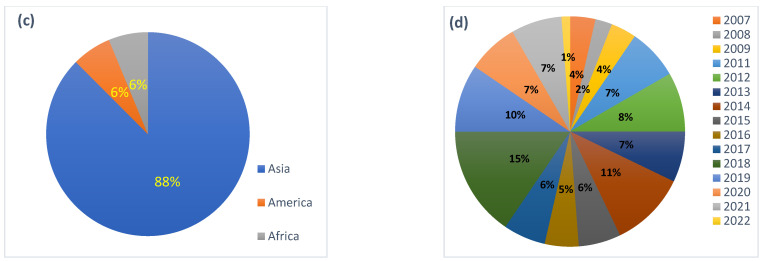
The percentages of studies conducted in the field of antibiotics in (**a**) seas, (**b**) rivers, and (**c**) lakes on different continents, and (**d**) the percentages of studies conducted during different years.

The Web of Science database was used to find published articles related to the present study. Additionally, for a specialized search, keywords such as ‘Antibiotics + Marine environment’, ‘Antibiotics + Sediment’, ‘Live creatures + Antibiotics’, ‘Antibiotic + river + lake + marine + Sea + sediment + organism + water body’, and ‘Review Antibiotics + microorganism + biota’ were used. There were 99 articles on antibiotics in sediments, water, and biota in the environments (aquatic environments), as well as several review studies. To draw the figures, first, the studies were categorized by year, parts of aquatic environments, country, and continent, and then the graphs related to each item were drawn using Excel software. Figure 1a–c show the percentages of studies conducted on antibiotics in marine environments, rivers, and lakes on different continents. The figure shows that the majority of studies for all water resources were conducted in Asia. Figure 1d represents the percentages of studies conducted in different years. According to this figure, more studies in the different environments were conducted in 2018 than in any other year. After the reviews, the information of some articles (e.g., [9,17,18,19]) was found to be more complete than others, and we considered these articles for summarization. To determine the distribution of antibiotics in different aquatic environments, the geographical coordinates (i.e., longitude and latitude) mentioned in the articles and the concentrations of available antibiotics (sediment: ng/g, water: ng/L) in each study were collected; then, using the Arc Geographic Information System (ArcGIS) 10.3, the map of distribution was prepared.

## 3. Results and Discussion

### 3.1. Classification of Antibiotics

According to their chemical structures, antibiotics are classified into several groups, including macrolides, beta-lactams, tetracyclines, quinolones, fluoroquinolones, sulfonamides, phenicols, and penicillin [14]. The tendency of antibiotics to be present in different environments—such as water, soil, and the atmosphere—depends on their physicochemical properties, octanol/water dividing coefficient (K_ow_), distribution coefficient (K_d_), separation constants (pK_a_), vapor pressure, and Henry’s law constant (K_H_) [27]. Antibiotics such as penicillin are easily decomposed in the environment. At the same time, fluoroquinolones and tetracyclines are more stable, so they can exist for longer in the environment, spread more, and eventually accumulate at higher concentrations [6]. The presence of beta-lactam rings in the structure of amoxicillin causes its degradation in the environment. Ciprofloxacin and erythromycin are also resistant to degradation because they lack beta-lactam in their structure [28]. Fluoroquinolones and sulfonamides are the most dangerous antibiotics in the environment; however, these antibiotics may be degraded by sunlight [29]. Accumulation of nitrite and nitrogen oxide (a strong greenhouse gas) in aquatic environments due to possible processes of nitrification and denitrification is among the effects of these two antibiotics (fluoroquinolones and sulfonamides) on the environment [30]. Antibiotics can be classified into different categories based on their chemical structure and function, such as beta-lactams (BLs), fluoroquinolones (FQs), macrolides (MLs), sulfonamides (SFs), tetracyclines (TCs), etc. Table 1 summarizes the physicochemical properties of the most common antibiotics. An overview of the physicochemical properties of common antibiotics is briefly presented below.

Macrolide (ML) antibiotics form a group of 12–16 organ lactone rings that are replaced with one or more sugars (amino sugars). Other characteristics of this group include being lipophilic, having low solubility in water, and being weakly acidic. Macrolides are generally bacteriostatic; however, some of these drugs may be bactericidal at very high concentrations [33,37]. This group is often used to treat infections in the respiratory tract, skin, and soft tissue [38].

Beta-lactam antibiotics include a wide range of molecules that contain at least one beta-lactam ring in their molecular structures. These drugs are active against many Gram-positive, Gram-negative, and anaerobic organisms, interfering with the cell wall synthesis of reproducing bacteria [39]. The presence of a typical four-membered (β)-lactam ring in the structure of this group gives them unstable thermal properties [40]. Beta-lactams, including penicillin—which accounts for 50–70% of antibiotics—are the most widely used antibiotics [41].

Sulfonamides (SAs) are derived from a p-amino-benzene-sulfonamide functional group. This group has acidic and basic properties [42]. These antibiotics are among the most widely used antibiotics in the world. The decomposition half-life of these antibiotics under light and heat is more than one year. They also have a relatively low adsorption capacity to solid matrices compared to other antibiotics. In addition, other applications of this type of antibiotics are as corrosion inhibitors and in the production of polymers. The most frequent use of these antibiotics is in veterinary medicine [33].

Tetracyclines are one of the main groups of antibiotics used for veterinary and human medicine, agriculture, and as food additives to enhance the growth of animals. These are amphoteric and degradable antibiotics that are unstable in bases but stable in acids. These antibiotics cause severe environmental problems and serious damage to human health. Since conventional WWTPs are not able to fully eliminate these micropollutants, the removal efficiency of tetracyclines in treatment plants has been reported between 12 and 80% [33,43]. Studies have shown that approximately 25–75% or 70–90% of tetracyclines used for treating animals enter the environment through urine and feces [44].

The quinolones’ properties are fat-solubility and resistance to acidic hydrolysis, alkalinity, high temperatures, and ultraviolet radiation damage. This group has many applications, including the treatment of infectious diseases and the promotion of livestock and aquaculture. They enter aquatic environments through untreated human and animal wastewater or direct discharge from aquaculture products [45].

### 3.2. Occurrence of Antibiotic Pollution in Seawater

**Figure 2:**
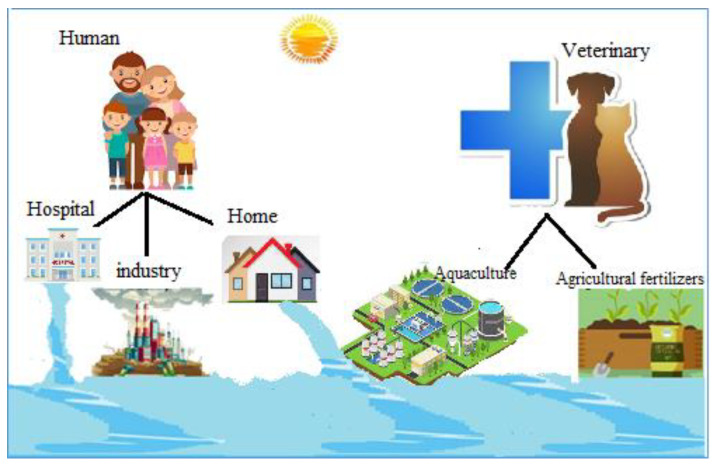
Means of entry of antibiotics into aquatic environments.

Over the past decades, the presence of antibiotics in the environment has created concerns around the world. Antibiotics are widely used in human and veterinary medicine and enter the environment through different pathways. Figure 2 shows the routes of entry of antibiotics into aqueous media. A significant portion of antibiotics enter marine environments through effluents from wastewater treatment plants and river inlets [46]. Sulfamethoxazole, azithromycin, and ciprofloxacin are some antibiotics used by humans, and their residues have been traced in the environment. Sulfamethoxazole, trimethoprim, azithromycin, and enrofloxacin are converted into ciprofloxacin in living organisms and are the most commonly used antibiotics for biota [34].

**Figure 3:**
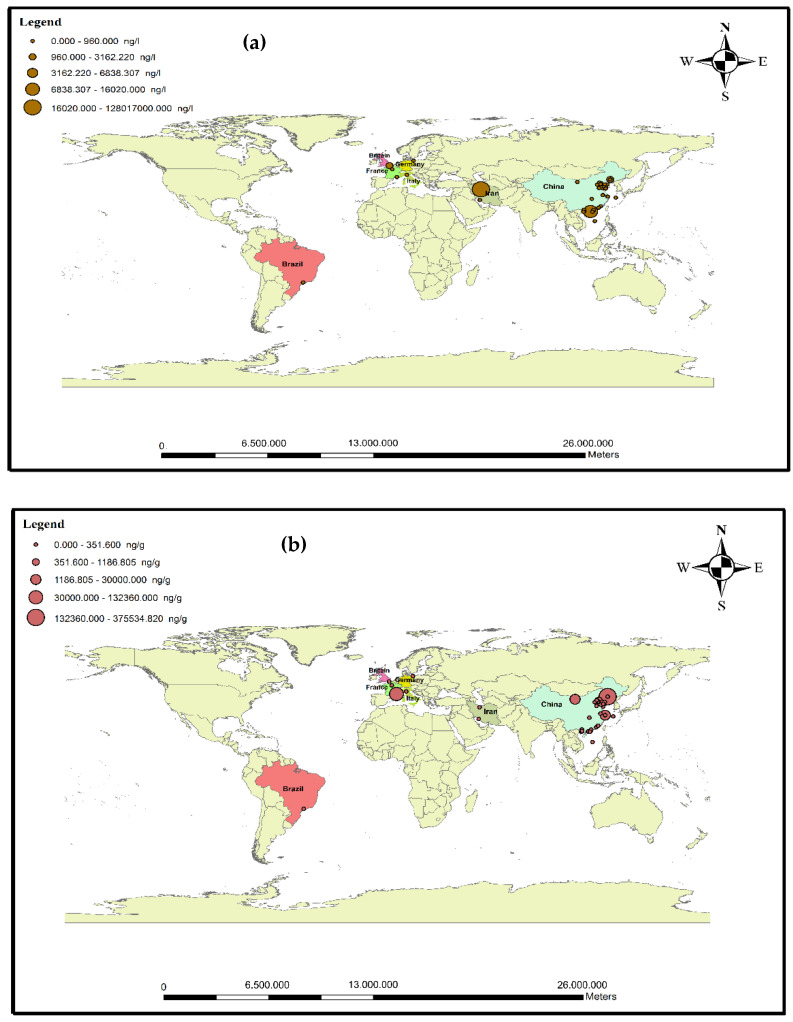
Map of antibiotic pollution in (**a**) water and (**b**) sediments of seas around the world.

The presence of antibiotics in the sludge and effluent of municipal treatment plants, hospitals, industrial centers, and livestock farms has resulted in the occurrence of pollution in marine environments, surface water, soil, and groundwater [47]. Consumed drugs and their metabolites are introduced into natural ecosystems through excretion (i.e., urine and feces) after a short time in the bodies of humans/animals [48,49]. The stability and accumulation of antibiotics in marine environments can endanger human health [28]. Chen et al. declared that about 92,700 tons of antibiotics was used in China, of which 53,800 tons was released into the environment. According to these results, it can be concluded that antibiotics are present in surface waters, groundwater, and coastal waters [15]. The presence of these compounds in water resources has become one of the most important public health concerns. Many studies have been conducted to determine the fates and effects of antibiotics in aquatic environments [50]. In marine environments, the concentrations of antibiotics in cold seasons are higher than in warm seasons [51]. The continued entry of antibiotics into the environment may pose potential risks to ecosystems and humans through food chains. Studies in the European Union have shown that ciprofloxacin (CIX), ofloxacin (OFX), erythromycin (ETM), and sulfadiazine (SDZ) can pose high environmental risks to aquatic life [10]. More than 100 types of antibiotics are used by humans and other living organisms. So far, 70 antibiotics have been observed in surface waters and sediments [47]. Figure 3a,b show the distribution of antibiotics in seawater and sediments in marine areas worldwide. According to Figure 3a, the waters most contaminated with antibiotics were found in Iran and China.

The antibiotics SMT (15–328 ng/L), SMZ (20–174 ng/L), and TMP (4–7 ng/L) had the highest concentrations among the 12 studied antibiotics in Vietnamese waters [52]. In a study by Chan et al., SFs, MLs, FQs, and TMP ng/L were identified as the most abundant antibiotics in the aquatic environment [53]. Kafaei et al., during their 2018 study in the Persian Gulf, concluded that among the available antibiotics, the concentration of NOR was 1.21–51.50 ng/L [50]. Zhang et al. reported that the antibiotics with the highest frequencies were DETM (71.6 ± 276 ng/L), NOX (1.56 ± 1.46 ng/L), and ENX (0.85 ± 0.65 ng/L) [54]. In the Yellow Sea of China, it was concluded that the antibiotics ENR and CIP had the highest frequency in seawater samples, at concentrations of 0.56–125.96 ng/L and 14.94–48.26 ng/L, respectively [51]. Table 2 shows the abundance of antibiotics in seawater and sediments in the evaluated seas around the world.

### 3.3. Occurrence of Antibiotics in Sediments

Sediments are considered to be reservoirs of pollution and receive large amounts of organic pollutants [15]. Antibiotics enter aquatic environments through effluents of agricultural and aquaculture activities and eventually reach sediments. The concentrations of antibiotics in sediments that are exposed to the entry of the aforementioned effluents are higher than in other areas [16]. Sediments act as reservoirs of antibiotics due to their high absorption capacity. Contaminants such as heavy metals and antibiotics can be absorbed by the sediment and cause antibiotic resistance [50]. The concentrations of antibiotics in sediments depend on seasonal changes, water flow, sediment characteristics, and the amounts of antibiotics used in the area [67]. Microbial activity in sediments can reduce the concentrations of antibiotics over time. However, some antibiotics or their metabolites can remain in the environment for a long time, depending on the conditions. Many antibiotics can bind to sediment particles, and only a tiny amount are bioactive [68]. Many studies have also shown that the absorption of antibiotics into sediments is influenced by changes in the flow and volume of water and its physicochemical properties (e.g., water temperature and the pH of water and sediments) [69]. Based on previous studies, the mobility of antibiotics increases in the presence of dissolved organic carbon and colloidal matter [70,71]. Kafaei et al. (2018), Zhao et al. (2016), and Cheng et al. (2016) have stated that the uptake of antibiotics by sediments is influenced by the pH and the contents of clay, silt, organic matter, and ionic matter of sediments [50,56,72]. Sediments are an ideal environment for the accumulation and propagation of antibiotic-resistance genes [73]. To determine quantitative relationships between antibiotics in the sediment and water phases, a quasi-partitioning coefficient (k_d_, s) is used. However, because there is no dynamic adsorption between sediment and water, this value cannot be considered an accurate partition coefficient [56]. Negligible concentrations of antibiotics that are soluble in water have been reported in sediments [52]. Factors such as the absorption or excretion of antibiotics over time and increasing ambient temperature reduce the concentrations of antibiotics in aquatic environments and, ultimately, sediments [74]. High concentrations of quinolones in sediments can be attributed to their chelation with cations and binding with particulate matter, delaying their degradation [69]. Other factors related to high concentrations of quinolones in sediments include a high dividing coefficient, low solubility, and low biodegradation [15]. The concentrations of eight antibiotics (four sulfonamides and four tetracyclines) in the sediment samples of two lakes in China were reported to be 117.97 ng/g (TCs) and 77.73 ng/g (SFs) in the sediment of East Dongting Lake and 1519.40 ng/g (SFs) and 126.27 ng/g (TCs) in the Lake Honghu sediment [73]. Zhang et al. concluded that fluoroquinolones and tetracyclines at concentrations of 12 ng/g and 11.8 ng/g, respectively, were the most abundant antibiotics in the evaluated sediment samples [75]. Arikan et al. reported that the concentration of the antibiotic clarithromycin in the sediment samples of the studied area was 1–180 ng/g, and it was the most abundant antibiotic [76]. Antibiotics studied in sediments in other parts of the world are summarized in Table 2. According to the sediment columns in Table 2, Table 3 and Table 4, it can be stated that the average concentrations of fluoroquinolones in sediments of seas, rivers, and lakes are 431.06 ng/g, 1020.58 ng/g, and 167.74 ng/g, respectively. Since sediments act as reservoirs of various pollutants, the concentrations of antibiotics in sediments are higher than those in water (except for lakes).

### 3.4. Occurrence of Antibiotics in Rivers

**Figure 4:**
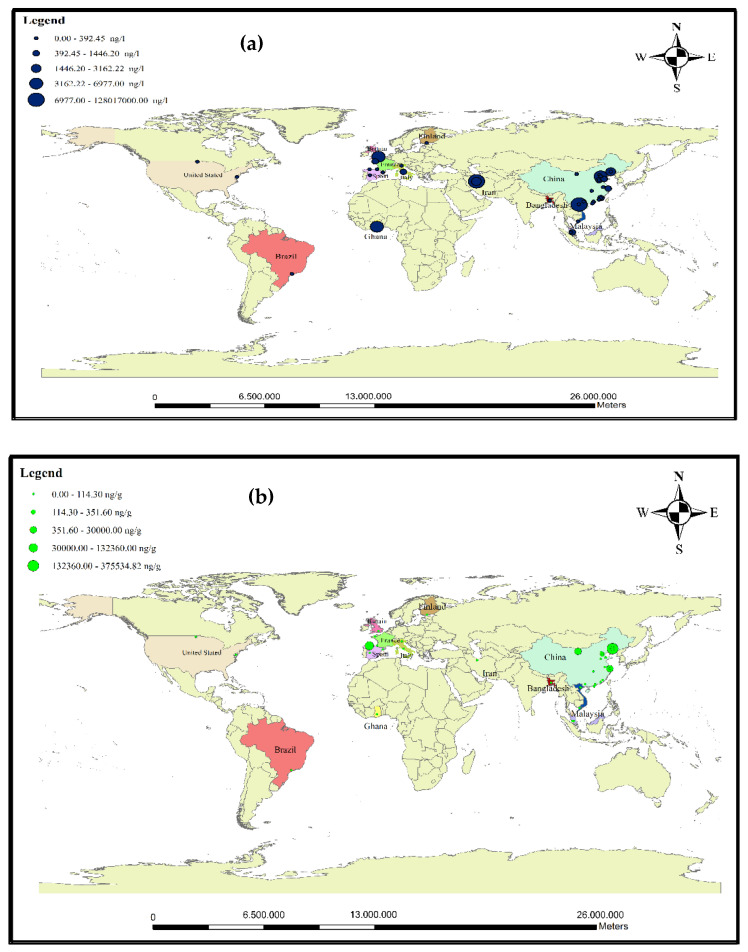
Map of study areas in (**a**) water and (**b**) sediments of rivers around the world.

Investigating the occurrence of antibiotics in aquatic environments helps us to assess their potential threat to ecosystem balance [104]. Antibiotic contamination in small stream-like rivers is mainly due to wastewater discharge [28]. Many rivers face serious problems due to antibiotic contamination. The presence of antibiotics in aquatic environments causes the accumulation of these contaminants in biota [69]. In recent years, antibiotics have come to the fore because of their potential threat to aquatic ecosystems and public health in river systems [15]. Various antibiotics have been frequently identified in surface water, groundwater, and drinking water. High concentrations of antibiotics in rivers also affect human populations, living organisms, and water flows [67]. The concentrations of pollutants (including antibiotics) in rivers passing through urban and rural areas have been increasing, which is caused by the discharge of sewage into these water sources. Moreover, population density downstream of the river compared to upstream, dehydration, and insufficient continuous flow of seasonal runoff to dilution are the main factors of high antibiotic concentrations in the lower parts of the rivers [125]. Studies on the cognition and mechanisms of action of antibiotics in humans show that their function is different in fish, algae, birds, and other species that live in rivers [126]. Concentrations of antibiotics in rivers that are exposed to effluent from sewage are higher than in those that are not exposed to effluent [52]. Estuarine sediments act as reservoirs for antibiotics and potential sources of secondary contamination that are affected by changes in environmental conditions [83]. Reducing the concentrations of antibiotics in rivers can be linked to dilution effects. Variable concentrations in wastewater and effluents are due to the chemical transformation of pollutants—which become metabolites—along with purification systems that have little impact in removing antibiotics [102,127]. Many studies have been performed to evaluate antibiotics in rivers around the world. Table 3 shows the concentrations of antibiotics in the sediments and water of rivers around the world. According to a study on a river in France, the concentrations of sulfamethizole, norfloxacin, and trimethoprim were 544 ng/L, 163 ng/L, and 45 ng/L, respectively [104]. Wang et al., while examining river waters in China, concluded that the concentrations of doxycycline, oxytetracycline, and tetracycline were 56.09 ng/L, 18.98 ng/L, and 11.16 ng/L, respectively [93]. In 2019, the mean amoxicillin concentration in the effluent of WWTPs in Italy was reported to be 1258 ± 7.6 ng/L [112]. The authors have stated that if these pollutants enter the rivers, they can create a high ecological risk. Figure 4a,b show the distribution of antibiotic concentrations in the water and sediments of rivers worldwide. Based on data analysis of published articles, it can be stated that the average concentration of sulfonamides in rivers is 191.11 ng/L. The high concentrations of sulfonamides can be attributed to the widespread use of these antibiotics and their relatively high stability (one year).

### 3.5. Occurrence of Antibiotics in Lakes

**Figure 5:**
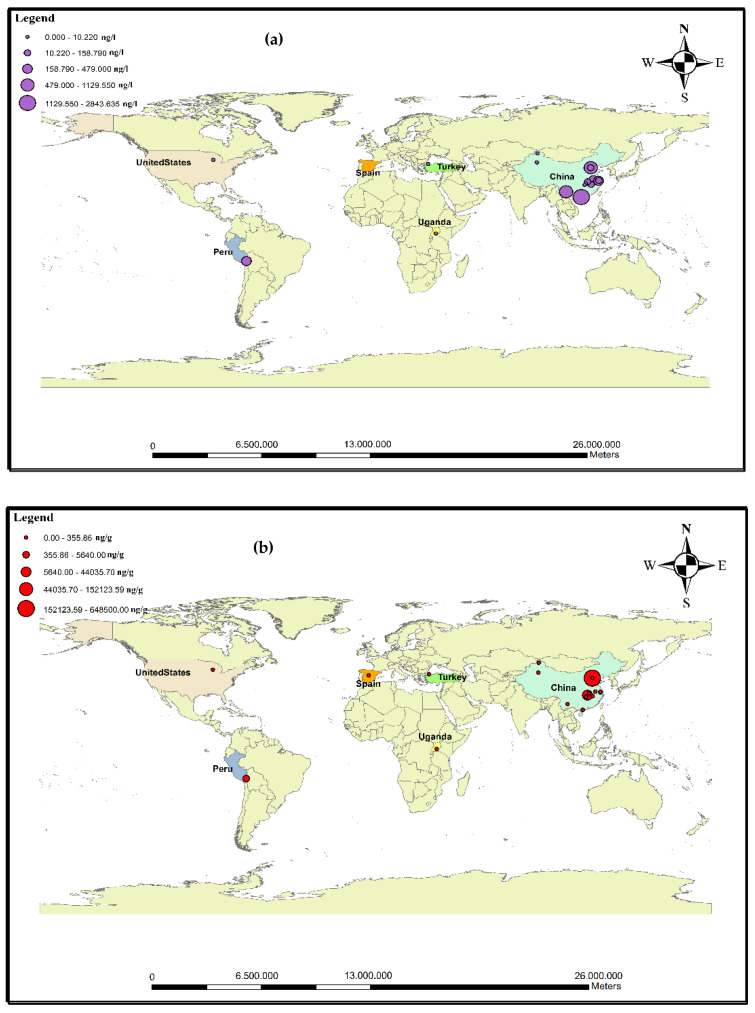
Map of studied areas for antibiotic contamination in (**a**) water and (**b**) sediments of lakes around the world.

As the use of antibiotics is increasing in industrialized and developing countries, it has led to the identification of these contaminants in surface waters, sediments, and biota around the world. The entry of antibiotics into aqueous media takes place through several sources. Most wastewater treatment plants are not able to effectively remove antibiotics, so their output can contaminate surface waters with antibiotics [16]. The antibiotic-laden effluents from agriculture enter environments such as rivers and lakes. Lakes, unlike rivers with their high water exchange, have low water circulation and, therefore, are more exposed to antibiotic contamination [73]. Although lakes have high potential for long-term storage of antibiotics, information on antibiotic contamination in lakes is much scarcer than in rivers. The entry of effluents from human and aquaculture activities increases the concentrations of these pollutants in lakes [128]. The accumulation of metals and antibiotics in urban and rural lake sediments is a serious threat to public health due to their potential for antibiotic resistance [7]. Among the various antibiotics, quinolones can bind to particulate matter in lakes because they are susceptible to optical degradation, while the biodegradation of macrolides has caused them to exist at low levels in surface water [69]. Table 4 shows the studies on antibiotic contamination in the sediments and water of lakes around the world. In a survey of a lake in Turkey, amoxicillin had a concentration of 1.1–1.15 ng/L [28]. In another study by Su et al., erythromycin, with a concentration of 7.26–99.22 ng/g, had the highest concentration in the sediments of the studied lake [57]. In the sediments of Lake Michigan, the antibiotics with the highest concentrations were azithromycin (147.28 ng/g), clarithromycin (67.66 ng/g), and ciprofloxacin (33 ng/g). Azithromycin (12.5 ng/L) and sulfamethoxazole (10.22 ng/L) also had the highest concentrations in Lake Michigan’s water [116]. Ciprofloxacin, norfloxacin, and ofloxacin, at concentrations of 75.5 ng/g, 55.2 ng/g, and 108.9 ng/g, respectively, were the most abundant antibiotics in the sediments of Dianchi Lake (China); moreover, sulfamethoxazole and ofloxacin, at concentrations of 17.6 ng/L and 713.6 ng/L, respectively, had the highest levels among antibiotics in the water samples of this lake [120]. Based on Table 4, it can be concluded that the average concentration of fluoroquinolones in the water of the studied lakes was 369.74 ng/L. According to the results of previous studies, it can be concluded that the concentrations of antibiotics in lake water are higher than in rivers and seas. This can be attributed to the stagnant flow of water in lakes and the continuous influx of agricultural, aquacultural, and human wastewaters containing antibiotic pollution. Figure 5a,b show the studies on the levels of antibiotics in the water and sediments of lakes. Based on this figure, the water and sediments of lakes in China and Peru have been more contaminated with antibiotics than in other parts of the world.

### 3.6. Occurrence of Antibiotics in Biota

**Figure 6:**
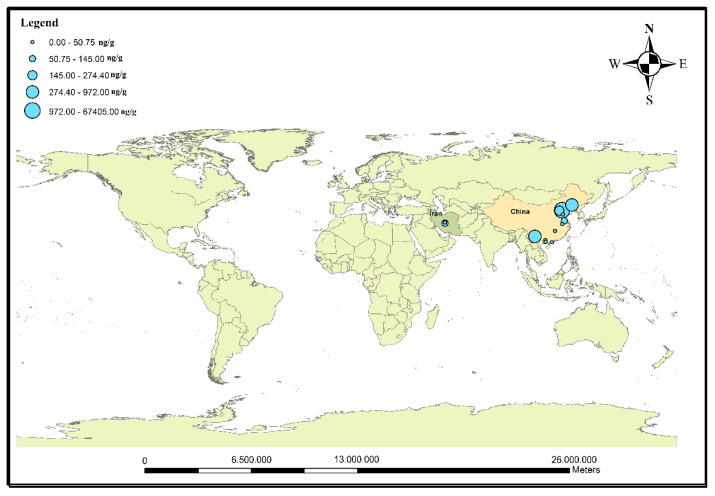
Antibiotic studies in biota in aquatic environments around the world.

Antibiotics are widely used to treat bacterial infections in humans and animals. Some are also used as growth stimulants in pigs [129] and plants [130]. The chemical structure of antibiotics is such that it causes a positive performance for the growth of some macrobiota [3,131]. The entry of these pollutants into aquatic environments from various sources causes biological accumulation and magnification in marine organisms. According to recent studies, contamination of water sources with antibiotics can double the toxic effects of cocktails in water (released from homes and commercial sectors) by creating more toxic compounds [132,133]. There is limited information on the potential for the bioaccumulation of antibiotics in organisms and food chains [69]. The bioaccumulation of antibiotics in marine organisms such as oysters is affected by several factors, including the degree of ionization and the K_ow_ partition coefficient. If the degree of ionization is not high and the K_ow_ partition coefficient is not between 2 and 6, the concentrations of antibiotics in oysters decrease [46]. Extensive and improper use of antibiotics causes large amounts of these contaminants in the tissues of aquatic organisms. The maximum virtual values in fish tissue for florfenicol (1000 µg/kg), total enrofloxacin and ciprofloxacin (100 µg/kg), oxytetracycline (100 µg/kg), tetracycline (100 µg/kg), chlortetracycline (100 µg/kg), and sulfonamide (100 µg/kg) were set by the European Commission [134]. Figure 6 presents the study of antibiotics in biota in aquatic environments worldwide. Most of the reported studies in this field have been implemented in China. Chen et al., during a study on fish (*Lutjanus russelli*, *Lutjanus erythopterus*, and *Trachinotus ovatus*), mollusks (*Atrina pectinata* Linnaeus, *Meretrix lusoria*, *Trisidos kiyoni*, and *Crassostrea rivularis* Gould), crabs (*Calappa philargius*), and shrimps (*Fenneropenaeus penicillatus*) in southern China, concluded that enrofloxacin was a high-concentration antibiotic in the muscles of the studied samples [46]. Sulfamethoxazole and norfloxacin were detected by Zhang et al. in a study on shrimps and tilapia [54]. In Bangladesh, sulfamethoxazole and trimethoprim had the highest concentrations in the studied organisms (i.e., finfish and shellfish) [88]. In another study conducted in Iran, enrofloxacin and fluoroquinolones were the predominant antibiotics in the farmed rainbow trout (*Oncorhynchus mykiss*) [135]. Table 5 lists the studies that reported the concentrations of antibiotics in biota in aquatic environments around the world. According to the information in the table, it can be concluded that among the different continents, the number of studies conducted in Asia was the highest. Among different antibiotics, the concentrations of fluoroquinolones were reported to be higher than those of other antibiotics. Furthermore, among different Asian countries, the average concentrations of fluoroquinolone were reported as 273.67 ng/g and 17.88 ng/g in China and Iran, respectively.

## 4. Conclusions and Remarks

The present study reviewed the reports of antibiotic concentrations in different parts of aquatic environments (i.e., seas, rivers, lakes, and marine organisms living in these environments). It can be concluded that among the different antibiotics, fluoroquinolones and sulfonamides had the highest concentrations in most of the studied environments. The average concentration of fluoroquinolones in sea, lake, and river sediments was 539.79 ng/g, while in sea and lake water it was 204.85 ng/L and 369.74 ng/L, respectively. Moreover, sulfonamides had the highest concentrations in rivers, with an average concentration of 191.11 ng/L. Antibiotics in aquatic tissues such as shrimp, fish, etc., can threaten the health of humans as consumers of these aquatic organisms. The average concentration of fluoroquinolone, as the most abundant antibiotic in marine organisms (fish, shrimp, oysters, fin whales, etc.), was reported as 145.77 ng/g. Among different countries around the world, the highest concentrations of antibiotics were reported in China. Furthermore, the results of the studies conducted in aquatic environments showed that the environments that are exposed to various effluents (urban, rural, veterinary, aquaculture, etc.) have higher concentrations and a greater variety of different antibiotics. Therefore, it is necessary to take the required approaches to reduce the entry of these pollutants into the environment. The following recommendations can be made for future investigations:

- Detect the concentrations of antibiotics in more aquatic organisms.
- The function of antibiotics is different in different organisms; therefore, it is necessary to study the concentrations of antibiotics in different organisms (fish, algae, etc.) simultaneously.
- Since antibiotics can accumulate in sediments of aquatic environments, it is recommended that future studies study the concentrations of antibiotics in sediments at different depths.
- The widespread use of antibiotics and the lack of advanced sewage treatment systems in developing countries have caused the pollution of water sources, necessitating more detection of antibiotic contamination and the improvement of treatment systems to remove pollutants.

## Author Contributions

Conceptualization, Z.M. and A.M.S.; methodology, T.M. and B.R.; software, M.S.; validation, S.F., B.R., T.N. and M.S.; investigation, Z.M. and T.N.; resources, T.M.; data curation, B.R.; writing—original draft preparation, B.R. and T.M.; writing—review and editing, M.S.; visualization, B.R.; supervision, M.S. All authors have read and agreed to the published version of the manuscript.

## Figures and Tables

**Table 1:** Physicochemical properties of the most common antibiotics.

Class	Antibiotics	Symbol	Molecular Formula	Molecular Weight	Solubility in Water (mg/L)	Main Use	Ref.
Tetracyclines (TCs)	Chlortetracycline Doxycycline Oxytetracycline Tetracycline	CTC DXC OTC TET	C_22_H_23_ClN_2_O_8_ C_22_H_24_N_2_O_8_ C_22_H_24_N_2_O_9_ C_22_H_24_N_2_O_8_	478.88 444.43 460.43 444.43	259 (25 °C) 50,000 (25 °C, pH = 2.16) 313 (25 °C) 231 (25 °C)	Veterinary Human, Veterinary Human, Veterinary, Plants Human, Veterinary	[31,32]
Sulfonamides (SFs)	Sulfadiazine Sulfamerazine Sulfamethazine Sulfamethizole SulfamethoxazoleSulfapyridine Sulfathiazole	SDZ SMR SMT SMZ SMX SPY STZ	C_10_H_10_N_4_O_2_S C_11_H_12_N_4_O_2_S C_12_H_14_N_4_O_2_S C_9_H_10_N_4_O_2_S_2_ C_10_H_11_N_3_O_3_S C_11_H_11_N_3_O_2_S C_9_H_9_N_3_O_2_S_2_	250.28 264.31 278.33 270.33 253.28 249.29 255.32	77 (25 °C) 202 (20 °C) 1500 (29 °C) 1050 (37 °C) 610 (37 °C) 268 (25 °C) 373 (25 °C)	Veterinary - Human, Veterinary Human Human - Veterinary	[33,34]
Macrolides (MLs)	Azithromycin Clarithromycin Erythromycin Roxithromycin Tylosin	AZI CLA ERY ROX TYL	C_38_H_72_N_2_O_12_ C_38_H_69_NO_13_ C_37_H_67_NO_13_ C_41_H_76_N_2_O_15_ C_46_H_77_NO_17_	748.98 747.95 733.93 837.05 916.10	2.37 (25 °C) 1.69 (25 °C) 4.2 (25 °C) 0.0189 (25 °C) 211	Human Human Human, Veterinary Human -	[8,34]
Fluoroquinolones (FQs)	Ciprofloxacin Enrofloxacin Levofloxacin Lomefloxacin Norfloxacin Ofloxacin	CIP ENR LEV LOM NOR OFL	C_17_H_18_FN_3_O_3_ C_19_H_22_FN_3_O_3_ C_18_H_20_FN_3_O_4_ C_17_H_19_F_2_N_3_O_3_ C_16_H_18_FN_3_O_3_ C_18_H_20_FN_3_O_4_	331.34 359.39 361.37 351.35 319.33 361.37	30,000 (20 °C) 612 1440 27,200 280 (25 °C) 10,800 (25 °C)	Human Veterinary - - - Human	[8,34,35]
β-Lactams	Amoxicillin Ampicillin Cephalexin Cefazolin Penicillin	AMO AMP CEP CEZ PEN	C_16_H_19_N_3_O_5_S C_16_H_19_N_3_O_4_S C_16_H_17_N_3_O_4_S C_14_H_14_N_8_O_4_S_3_ C_16_H_18_N_2_O_4_S	365.40 349.40 347.39 454.51 334.39	3430 (25 °C) 10,100 (21 °C) 10,000 210 (25 °C) 210	Veterinary Veterinary Human Human Veterinary	[8,34]
Other classes	Chloramphenicol Lincomycin Trimethoprim	CHP LIN TMP	C_11_H_12_C_l2_N_2_O_5_ C_18_H_34_N_2_O_6_S C_14_H_18_N_4_O_3_	323.13 406.54 290.32	2500 (25 °C) 927 (25 °C) 400 (25 °C)	Human Human Human	[8,36]

**Table 2:** Studies on antibiotics in seawater and sediments in marine environments around the world.

Sea (Country)	Geographical Coordinates	Sediments (ng/g)	Seawater (ng/L)	Number of Stations	Ref.
Beibu Gulf (China)	21°30′36.00′′ N, 108°07′07.60′′ E	-	SMX: 15.9, TMP: 4.11, and ERY: 2.59–47.6	4	[55]
Bohai Bay (China)	38°53′18.37′′ N, 119°48′40.09′′ E	TCs: 7.71–130.36	TCs: 41.53–222.43	16	[56]
Maowei Sea (China)	21°50′49.05′′ N, 108°29′54.87′′ E	-	DETM: 276 ± 71.6, NOX: 1.56 ± 1.46, and ENX: 0.85 ± 0.65	7	[54]
Beibu Gulf (China)	22°48′45.26′′ N, 108°22′18.46′′ E	FQs: 9.69–15.43	MLs: 52.9477.76	3	[57]
Hailing Island (south coast of England)	21°37′41.12′′ N, 111°55′00.56′′ E	ERY: 0.8–4.8	OTC: 16,000 and TMP: 20	6	[46]
North coast of the Persian Gulf (Iran)	28°55′09.27′′ N, 50°55′16.92′′ E	NOR: 1.40–25.32	NOR: 1.21–51.50	3	[50]
Spain	40°27′49.20.′′ N, 3°44′57.18′′ W	-	AMO: 326.70	-	[58]
Victoria Harbour (South China)	22°45′40.74′′ N, 51°24′56.21′′ E	OFL: 17.5, ERY: 17.5, and SMX: 0	SMX: 1.036–629	1	[59]
Baltic Sea (Northern Europe)	53°56′13.58′′ N, 14°11′59.69′′ E	TMP: 35.7 and OTC: 15.5	SMX: 311 and TMP: 279	29	[60]
Bohai Sea (China)	38°56′14.71′′ N, 121°12′22.67′′ E	-	ENR:139, SMX: 17.7, and TMP: 5.0	22	[61]
Hailing Bay (China)	50°46′37.34′′ N, 0°58′41.76′′ E	CIP: 184 and ERY < 1.95	ERY: 1318, CLA: 15.16, TMP: 4.24, and OTC: 2.12	5	[62]
Yellow Sea (China)	36°46′43.11′′ N, 117°53′14.95′′ E	OTC: 895.32–1478.29	ENR: 0.56–125.96 and CIP: 14.94–48.26	11	[51]
Xiong’an New Area (China)	38°43′ and 39°10′ N, 115°38′ and 116°20′ E	FQs: 38.03–406.31	AMO: 12.71–260.56 in surface water AMO: ND-196.12 in groundwater	-	[63]
Po Valley (Italy)	45°00′00.06′′ N, 10°30′00.79′′ E	-	CLA: 128,103 and CIP: 124	2	[64]
Jiaozhou Bay (China)	36°11′24.11′′ N, 120°18′02.07′′ E	OFL: ND −3.337	-	2	[15]
Bohai Bay(China)	37°41′04.55′′ N, 120°17′24.23′′ E	-	NOR: 460, OFL: 390, and CIP: 110	28	[65]
East China Sea	30°26′36.19′′ N, 125°57′35.29′′ E	FQs: 7.3, MLs: 5.2, and SFs: 2.6	β-lactams: 215.6, FQs: 54.2 and SF_S_: 39.3	3	[66]

**Table 3:** Studies on antibiotics’ concentrations in the sediments and water of rivers around the world.

River (Country)	Geographical Coordinates	Sediments (ng/g)	Water (ng/L)	Number of Stations	Ref.
Haihe River (China)	39°02′04.57′′ N, 117°27′55.19′′ E	NOR: 63.5, ENR: 50.8	SMX: 68	5	[77]
Liao River (China)	41°56′46.28′′ N, 122°51′06.01′′ E	MLs: 375,130, TCs: 404.82	MLs: 3,162.22	50	[78]
Jiulong River (China)	25°00′12.37′′ N, 117°32′02.54′′ E	-	SFs: 81.07	35	[79]
Huangpu River (China)	31°08′02.87′′ N, 121°27′17.68′′ E	TCs: 18,000, MLs: 12,000	SFs: 34–859	30	[80]
Huangpu River (China)	31°08′02.87′′ N, 121°27′17.68′′ E	-	SMT: 468.13 and TCs: 75.29	19	[81]
Yellow River (China)	36°31′08.14′′ N, 116°36′38.16′′ E	-	NOR: 327, OFL: 119, and MLs: 91	24	[82]
Pearl River Estuary (china)	22°46′10.36′′ N, 113°37′17.53′′ E	NOR: 7.62, OFL: 3.63, and: MLs: 2.69	NOR: 68.06, OFL: 6.93, and MLs: 21.7	14	[83]
Haihe River Basin (China)	39°02′12.42′′ N, 117°27′57.71′′ E	SFs: 210–385	OTC: (4.0 ± 0) × 10, ERY: (3.8 ± 0.6) × 10	15	[84]
Yangtze River (China)	29°43′.11.37′′ N, 112°39′01.61′′ E	-	ERY: 296	4	[85]
Pardo River (Brazil)	23°33′56.23′′ S, 46°45′26.60′′ w	-	TCs: ND ^a^	-	[86]
Lui River (Malaysia)	3°05′24.86′′ N, 102°25′55.60′′ E	-	AMO: ND- 4.44, 7.11–7.81, and 1.75–6.08, CIP: 52.50–138.17, 225.18–299.88, and 143.75–258.53, and SMX: 19.26–75.48, 96.81–109.34, and 84.31–114.24	3	[87]
Hai River (China)	15°29′17.12′′ N, 114°24′17.10′′ E	TCs: 2.76 × 10^2^	SMX: 1.57 × 10^2^ and TCs: 6.82 × 10^3^	5	[47]
Chaobai Rive (China)	40°01′43.21′′ N, 116°46′39.58′′ E	FQs: 12.0 and TCs: 11.8	SFs: 4.71–95.3 and MLs: 0.41–85.3	3	[75]
Rajshahi, Jessore and Mymensingh (Bangladesh)	23°36′30.76′′ N, 90°20′02.93′′ E	-	SMX: ND -20.02, TMP: ND -41.67 and SDZ: 17.97	6	[88]
Hanjiang River (China)	24°03′44.05′′ N, 116°30′10.98′′ E	SFs: 5.4, TCs: 9.6 and FQs: 5.4	SFs: 24, TCs: 10, and FQs: 5.5	14	[89]
Xiaoqing River (China)	37°16′34.19′′ N, 118°58′30.68′′ E	-	TMP: 1272, ERY: 97.36, and SMX: 76.84	10	[90]
Brahmaputra River (Bangladesh)	23°29′46.19′′ N, 90°22′22.79′′ E	-	TMP: 17.20		[91]
Jiulongjiang River (China)	25°00′13.52′′ N, 117°32′01.91′′ E	-	Average SFs: 383, TCs: 424.25, and FQs: 3.91	19	[92]
Yangtze River (China)	29°43′12.47′′ N, 112°39′00.51′′ E	-	DXC: 56.09, OTC: 18.98, and TET: 11.16	28	[93]
Beiyun River (China)	39°47′36.23′′ N, 116°47′42.81′′ E	-	ERY: 319	34	[88]
Yodo River (Japan)	34°45′23.69′′ N, 135°33′44.37′′ E	-	CLA.	4	[94]
Yangtze River (China)	29°43′12.47′′ N, 112°39′00.51′′ E	OFL: 8.4	ERY: 0.29	29	[95]
Yongjiang River (China)	22°47′49.95′′ N, 108°22′55.48′′ E	SMX: 0.032, SDZ: 0.017 and TMP: 0.32	SMX: 9.96, SDZ: 55.8, and TMP: 93.5	35	[96]
Alpine rivers	45°55′35.02′′ N, 11°34′45.04′′ E	-	TMP and SMX >100	12	[97]
Pearl River Delta (China)	21°30′N, 113°00′ E	-	Average concentrations OFL, SMX, and ERY: 1.2–127	8	[98]
Zhuhai City (China)	22°16′36.11′′ N, 113°35′06.26′′ E	AGs: 74.5–152	AG_S_: 54.6–134 and FQs: 154–256	9	[15]
Klang (Malaysia)	3°04′33.74′′ N, 101°37′27.20′′ E	-	AMO: 102.31	12	[99]
Nanjing (China)	32°04′19.29′′ N, 118°47′32.17′′ E	-	SFs: 23.52–219.00 and NOR: 146.72–290.20	13	[100]
Liao River in Jilin Province (China)	41°57′16.22′′ N, 122°51′26.12′′ E	OFL: 152.2 ± 108.3, OTC: 149 ± 147.6, and NOR: 62.8 ± 83.3	OTC: 266.9 ± 174.9, ERY: (103.2 ± 95.5, and OFL: 67.1 ± 77.3	-	[101]
Rivers of Tehran (Iran)	35°45′40.74′′ N, 51°24′56.21′′ E	-	AMO: 128,017,000,000 in 1000 people per day	2	[102]
Laizhou Bay (China)	37°18′38.00′′ N, 119°21′47.54′′ E	-	ENR: 209, CIP: 66 and TMP: 1.3–330	10	[103]
Seine River (Northern France)	48°35′44.76′′ N, 2°27′16.16′′ W	-	SMZ: 544, NOR: 163, and TMP: 45	5	[104]
Arc Rive (Southern France)	43°30′37.36′′ N, 5°28′22.27′′ w	AZI: 130,660 and CLA: 1700	CLA: 0.71	3	[105]
Cache La Poudre (United States)	40°25′11.32′′ N, 104°40′28.79′′ E	TCs: 6900–24,300, STZ: 4800, and RTM: 2100	SMX: 110, SFs: 110, and TCs: 20–180	5	[106]
Red River (Canada)	48°22′52. 85′′ N, 97°05′21.9′′ W	-	SMX: 1.5–7.6	-	[107]
Yellow River, Hai River, and Liao River (Northern China)	36°31′08.14′′ N, 116°36′38.16′′ E 39°02′12.42′′ N, 117°27′57.71′′ E 41°56′46.28′′ N, 122°51′06.01′′ E	NOR: 7.76, OFL: 3.49, and ERY: 8.11	-	3	[67]
Red River (Vietnam)	21°01′41.29′′ N, 105°50′03.30′′ E	-	SFs, MLs, FQs, and TMP	5	[53]
Kumasi (Ghana)	6°39′55.38′′ N, 1°36′58.58′′ W	-	SMX: 2861, ERY: 10.614–7944,	7	[108]
Finland	60°18′55.84′′ N, 24°53′47.17′′ E	-	CIP: 20	1	[109]
Mekong Delta (Vietnam)	10°05′36.74′′ N, 105°23′15.78′′ E	-	SMT: 15–328, SMZ: 20–174, and TMP: 7–44	6	[52]
Choptank River (USA)	38°40′51.80′′ N, 75°57′05.30′′ W	CLA: 11–34	CLA: 1–180	22	[76]
Red River (Northern Vietnam)	20°55′22.82′′ N, 105°58′11.95′′ E	-	SMT: 475–6662, SMX: 612–4330, ERY: 154–2246, and CLA: 2.8–778	10	[12]
Kan River, Firozabad Ditch, and location of Ekbatan WWTP and south Tehran (Iran)	35°45′10.60′′ N, 51°19′49.09′′ E	-	CIP: 552.6–796.2 in effluent, CIP: 127–248, and CEP: 523.3–977.7	22	[110]
Baix Fluvià (northeastern Catalonia, Spain)	41°32′35.50′′ N, 1°31′35.16′′ E	-	CIP: 211.8, SMX: 8.5	4	[111]
Rome (Italy)	41°54′20.31′′ N, 12°29′29.24′′ E	-	AMO: 1258 ± 7.6	4	[112]
Thames (UK)	51°33′58.29′′ N, 0°41′39.52′′ W	-	CLA: 5000, ERY: 790 and AZI: 7.3	33	[113]
Tagus (Spain)	39°42′20.03′′ N, 5°16′31.52′′ W	-	TMP: 4.42–99.89, SMX: 9.42–27.5, and AZI: 5.06–8.23	16	[114]
Adour Estuary (France)	43°37′01. 91′′ N, 1°26′43.21′′ W	-	NOR, OFL, and CIP	-	[115]

^a^ ND: non-detectable.

**Table 4:** Studies on antibiotics in the sediments and water of lakes.

Lake (Country)	Geographical Coordinates	Sediments (ng/g)	Water (ng/L)	Number of Stations	Ref.
Honghu Lake (China)	29°39′44.58′′ N, 113°20′52.91′′ E	TETs: 117.970, SAs: 77.730, and DC: 43,840	-	14	[73]
Dongting Lake (China)	28°59′37.73′′ N, 112°43′41.52′′ E	SAs: 151,940, TC: 126.270, and SMX: 57.320	-	14	[73]
Michigan Lake (China)	43°31′14.91′′ N, 87°13′19.25′′ W	AZI: 147.28, CLA: 67.66	SMX: 10.22	7	[116]
Dongting Lake (China)	28°49′38.33′′ N, 112°41′44.29′′ E	-	TMP: ND ^a^	42	[117]
Chaohu Lake (China)	31°33′31. 16′′ N, 117°34′27.24′′ E	-	SMX: 95.6, OFL: 383.4	8	[118]
Poyang Lake (China)	29°08′20.44′′ N, 116°11′39.41′′ E	-	SDZ: 56.2, OTC: 48.7, and DXC: 39.7	4	[119]
Dianchi Lake (China)	24°48′46.92′′ N, 102°41′20.88′′	CIP: 75.8, NOR: 55.2, and OFL: 108.9	SMX: 17.6–499.2, and OFL: ND-713.6	27	[120]
Taihu Lake (China)	31°26′.56′′ N, 120°23′.46′′ E	-	SMX: 7.24–53.59, NOR: 15.83–56.22, and OFL: 7.45–17.01	8	[121]
Turkey	41°03′14. 63′′ N, 28°33 ′25.75′′ E	-	AMO: 1.1–1.15	6	[28]
Taihu Lake (China)	31°14′.43.36′′ N, 120°12′ 13.35′′ E	OTC: 52.8, TC: 47.9	OTC: 47.8, SMT: 252.7	30	[16]
Baiyangdian Lake (China)	38°53′08.18′′ N, 116°00′48.19′′ E	NOR: 274.76, OFL: 39.73, TC: 25.71, and OTC: 15.66	TC: 25.95–31.60, OTC: 18.86–23.80	6	[74]
Baiyangdian Lake (China)	38°53′08.18′′ N, 116°00′48.19′′ E	FQs: 65,500–1,166,000	SFs: 0.86–1563	30	[69]
Bosteng Lake (China)	42°00′09.72′′ N, 87°01′40.97′′ E	CIP: 21.18–213.38, OFL: 18.39–94.1, and OTC: 4.61–20.67	-	-	[122]
Ulungur Lake (China)	47°17′53.10′′ N, 87°17′17.15′′ E	LOM: 6.34–53.85, CIP: 2.56–28.65, SAAM: 1.45–5.38, and SDZ: 1.03–3.68	-	-	[122]
Titicaca Lake (South America)	15°55′45.95′′ S, 69°20′07.44′′ W	TMP: 5000 SMX: 640	TMP: 130 SMX: 159	4	[123]
Hubei Province (China)	30°35′04.61′′ N, 114°18′23.95′′ E	-	OFL in the pond: 1. 15.98 and OFL in the pond: 2. 21–127.40	2	[15]
Nakivubo wetlands and Lake Victoria, Kampala (Uganda)	00°18′ N, 32°38′32′ E	CIP and Metronidazole: ND		8	[124]
Maoming City (China)	21°39′44.91′′ N, 110°55′33.35′′ E	ERY7: 26–99.22	In surface water ERY: 782–2634 and SDZ: 1.42–19.83; in the pond ERY: 19.02–2231	1	[57]

^a^ ND: non-detectable.

**Table 5:** Antibiotics reported in biota around the world.

Region (Country)	Geographical Coordinates	Antibiotics in Biota (ng/g)	Number of Stations	Ref.
Hailing Island (China)	21°37′41.12′′ N, 111°55′00.56′′ E	ENR: 16.6–31.8	6	[46]
Beibu Gulf (China)	22°48′45.26′′ N, 108°22′18.46′′ E	FQs: 0.68–4.75	3	[10]
Maowei Sea (China)	21°50′49.05′′ N, 108°29′54.87′′ E	NOX and SMX	7	[54]
Central, northern, western, and north-western Iran	32°25′40.46′′ N, 53°41′16.99′′ E	ENR:0.02–0.34 and FQs: 0.210	14	[135]
Central, northern, western, and north-western Iran	32°00′40.60′′ N, 53°41′16.99′′ E	FQs: 6.75–99.8 and SFs: 4.03–90.4	138	[134]
Baiyangdian (China)	38°53′08.18′′ N, 116°00′48.19′′ E	FQs:17.8–167, and MLs: 182	30	[69]
Dianchi lake (China)	24°48′46.92′′ N, 102°41′20.88′′ E	OFL: ND ^a^-713.6, SMX: 17.6–499.2	27	[120]
Liao River (China)	41°56′46.28′′ N, 122°51′06.01′′ E	FQs: 286.6–1655	50	[78]
Dongting Lake (China)	27°49′.38.07′′ N, 113°41′41.68′′ E	SDX: 1.06, ENR: 1.05, SDZ: 0.68 and SMT: 0.57	3	[136]
Hongze Lake (China)	33°17′.15.36′′ N, 118°43′23.90′′ E	OTC: 74, SDZ: 47, and CIP: 24	30	[137]
Haihe River (China)	39°02′04.57′′ N, 117°27′55.19′′ E	NOR: 500–51,600, CIP: 1670–12,500 and SMZ: 540–6,8000	5	[77]
Yellow Sea (China)	36°46′43.11′′ N, 117°53 ′14.95′′ E	CIP: 9.26 and ENR: 24.75	11	[51]
Cox’s Bazar, Shatkhira and Khulna (Bangladesh)	23°43′ 50.76′′ N, 90°21′21.22′′ E	SMX: ND-16.67 and TMP: 11.39	6	[88]

^a^ ND: non-detectable.

## Data Availability

Not applicable.
